# New Approach to Addison Disease: Oral Manifestations Due to Endocrine Dysfunction and Comorbidity Burden

**DOI:** 10.3390/diagnostics12092080

**Published:** 2022-08-28

**Authors:** Narcis Mihăiţă Bugălă, Mara Carsote, Loredana Elena Stoica, Dana Maria Albulescu, Mihaela Jana Ţuculină, Smaranda Adelina Preda, Ancuta-Ramona Boicea, Dragoș Ovidiu Alexandru

**Affiliations:** 1 Department of Medical Informatics and Biostatistics, Faculty of Medicine, University of Medicine and Pharmacy of Craiova, 200349 Craiova, Romania; 2 Department of Endocrinology, Carol Davila University of Medicine and Pharmacy, 050474 Bucharest, Romania; 3 C.I. Parhon National Institute of Endocrinology, Aviatorilor Ave. 34–38, Sector 1, 011683 Bucharest, Romania; 4 Department of Dermatology, Faculty of Medicine, University of Medicine and Pharmacy of Craiova, 200349 Craiova, Romania; 5 Department of Anatomy, Faculty of Medicine, University of Medicine and Pharmacy of Craiova, 200349 Craiova, Romania; 6 Department of Odontology, Faculty of Dental Medicine, University of Medicine and Pharmacy of Craiova, 200349 Craiova, Romania; 7 Department of Occupational Medicine, Faculty of Medicine, University of Medicine and Pharmacy of Craiova, 200349 Craiova, Romania

**Keywords:** Addison disease, cortisol, adrenal insufficiency, oral, tongue, periodontal disease, pigmentation, candidiasis, ACTH, autoimmune

## Abstract

This review highlights oral anomalies with major clinical impact in Addison disease (AD), including dental health and dermatologic features, through a dual perspective: pigmentation issues and AD comorbidities with oral manifestations. Affecting 92% of AD patients, cutaneomucosal hyperpigmentation is synchronous with or precedes general manifestations by up to a decade, underlying melanocytic infiltration of the basal epidermal layer; melanophages in the superficial dermis; and, rarely, acanthosis, perivascular lymphocytic infiltrate, and hyperkeratosis. Intraoral pigmentation might be the only sign of AD; thus, early recognition is mandatory, and biopsy is helpful in selected cases. The buccal area is the most affected location; other sites are palatine arches, lips, gums, and tongue. Pigmented oral lesions are patchy or diffuse; mostly asymptomatic; and occasionally accompanied by pain, itchiness, and burn-like lesions. Pigmented lingual patches are isolated or multiple, located on dorsal and lateral areas; fungiform pigmented papillae are also reported in AD individuals. Dermoscopy examination is particularly indicated for fungal etiology; yet, it is not routinely performed. AD’s comorbidity burden includes the cluster of autoimmune polyglandular syndrome (APS) type 1 underlying *AIRE* gene malfunction. Chronic cutaneomucosal candidiasis (CMC), including oral CMC, represents the first sign of APS1 in 70–80% of cases, displaying autoantibodies against interleukin (IL)-17A, IL-17F ± IL-22, and probably a high mucosal concentration of interferon (IFN)-γ. CMC is prone to systemic candidiasis, representing a procarcinogenic status due to Th17 cell anomalies. In APS1, the first cause of mortality is infections (24%), followed by oral and esophageal cancers (15%). Autoimmune hypoparathyroidism (HyP) is the earliest endocrine element in APS1; a combination of CMC by the age of 5 years and dental enamel hypoplasia (the most frequent dental complication of pediatric HyP) by the age of 15 is an indication for HyP assessment. Children with HyP might experience short dental roots, enamel opacities, hypodontia, and eruption dysfunctions. Copresence of APS-related type 1 diabetes mellitus (DM) enhances the risk of CMC, as well as periodontal disease (PD). Anemia-related mucosal pallor is related to DM, hypothyroidism, hypogonadism, corresponding gastroenterological diseases (Crohn’s disease also presents oral ulceration (OU), mucogingivitis, and a 2–3 times higher risk of PD; Biermer anemia might cause hyperpigmentation by itself), and rheumatologic diseases (lupus induces OU, honeycomb plaques, keratotic plaques, angular cheilitis, buccal petechial lesions, and PD). In more than half of the patients, associated vitiligo involves depigmentation of oral mucosa at different levels (palatal, gingival, alveolar, buccal mucosa, and lips). Celiac disease may manifest xerostomia, dry lips, OU, sialadenitis, recurrent aphthous stomatitis and dental enamel defects in children, a higher prevalence of caries and dentin sensitivity, and gingival bleeding. Oral pigmented lesions might provide a useful index of suspicion for AD in apparently healthy individuals, and thus an adrenocorticotropic hormone (ACTH) stimulation is useful. The spectrum of autoimmune AD comorbidities massively complicates the overall picture of oral manifestations.

## 1. Introduction

Adrenal insufficiency, either primary, known as Addison disease (AD), or secondary, represents a life-threatening condition, affecting 20–50 persons per 100,000 in Europe [1]. Depending on the acute or chronic presentation of AD, the clinical picture can include low blood pressure and collapse; unintentional weight loss; loss of appetite; chronic asthenia; nausea; vomiting; and abdominal, muscle, or joint pain [2]. 

A high adrenocorticotropic hormone (ACTH) level causes lingual and buccal anomalies of melanocytes in AD, which are responsible for hyperpigmentation [2,3]. An intraoral pigmentation might be the only sign of AD, and an acute event requiring a higher cortisol amount such as an illness or an accident might cause an acute form of adrenal insufficiency, while the patient is still associated with this single hallmark of the underlying condition [4]. Anomalies of oral pigmentation might not always reflect the level of disease control, but they represent a particular clue for clinicians of different specialties, and it should be noted that AD is a severe disease with a fatal outcome without adequate hormonal replacement. Additional oral manifestations are found in AD patients through the association of nonadrenal diseases such as autoimmune conditions or genetic syndromes such as autoimmune polyglandular syndrome 1 (APS) [5].

AD requires lifelong substitution with glucocorticoids (two or three times daily) and mineralocorticoids (once per day) [6,7,8]. Despite advances in therapy and adequate patient education, subjects with AD are associated with a higher cardiovascular risk than the general population and a more affected quality of life (QL) [9,10]. Prompt recognition is mandatory, while treatment of associated complications and comorbidities, including oral manifestations, improves the prognosis and overall QL [9,10]. Modern management of AD seeks to identify new regimes of glucocorticoid administration in order to improve associated morbidity and mortality [6,11].

**Figure 1:**
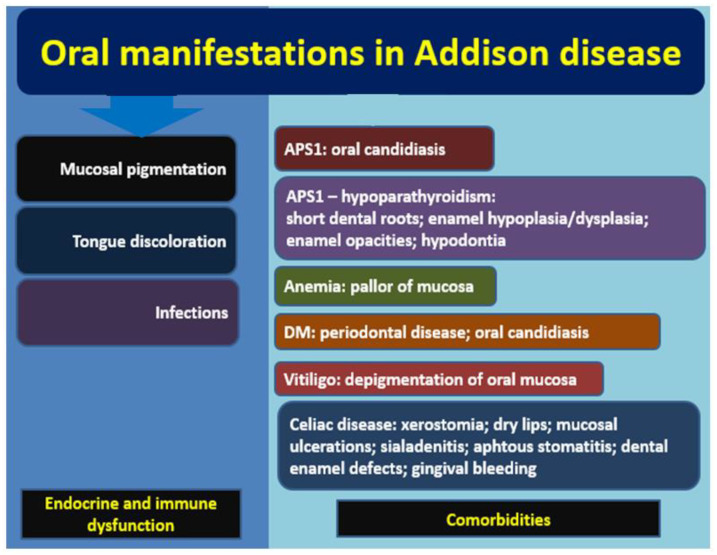
Overview of the main oral manifestations in Addison disease, either related to endocrine and immune dysfunction accompanying the disease itself (left side) or associated with a heterogeneous panel of comorbidities based on a common genetic or autoimmune background as seen in APS1, anemia, DM, vitiligo, and celiac disease (right side) (abbreviations: APS = autoimmune polyglandular syndrome; DM = diabetes mellitus) (see references in text).

This review highlights multidisciplinary aspects of AD, focusing on oral anomalies that might have a major clinical impact. The perspective includes dental health aspects and dermatologic features. We followed two main aspects of oral manifestations: pigmentation issues which are related to specific hormonal anomalies in AD, and potential oral lesions in AD patients, especially in autoimmune AD, that are induced by AD-related diseases with a common genetic background or similar autoimmune mechanisms (Figure 1).

## 2. Methods

The research was based on full-length, English literature, mostly PubMed papers, published between 2022 and 2012. The keywords of research were “Addison disease”, “adrenal insufficiency”, and “polyglandular autoimmune syndrome”, each in different combinations with “oral”, “dental”, “tongue”, “periodontal disease”, “candidiasis”, “diabetes”, “hypoparathyroidism”, “celiac disease”, “vitiligo”, “anaemia”, etc. We introduce two figures showing oral manifestations in AD; the patients agreed to the use of these images. 

### 2.1. AD and Mucosal Pigmentations

Proopiomelanocortin (POMC) is synthetized in corticotroph and melanotroph cells of the hypophyseal gland (anterior and intermediate lobes); POMC represents the subject of a tissue-specific post-translational cleavage that leads to active peptides such as ACTH which controls the adrenal cortex in addition to alpha-melanocyte stimulating hormone (MSH) or alpha-melanotropin that controls skin pigmentation through enhancing the dermal translocation of melanin granules and stimulation of melanogenesis [12,13]. Melanocortin receptors (MCRs) are responsible for these actions: MC1R is found in the skin, ACTH stimulates adrenal glucocorticoid production through MC2R activation, and MC3R and MC4R play different roles in several metabolic pathways (the role of MC5R is still incompletely described) [14]. On the other hand, hypothalamic corticotropin-releasing hormone (CRH) stimulates POMC gene expression in the pituitary gland, most of its actions being mediated by CRF1R and CRF2R receptors; CRH expression and its receptors in addition to POMC expression have also been identified in skin and mucosa, confirming a complex interaction between glucocorticoid axes and the cutaneomucosal system [15]. Conditions with POMC- and ACTH-associated pigmentation lesions include AD and Nelson’s syndrome (which develops in Cushing’s disease after bilateral adrenalectomy—typically a last option of therapy when all the other well-known methods have been unsuccessful), both of them underlying pituitary ACTH anomalies, while nonpituitary ACTH disorders include paraneoplastic (ectopic) Cushing’s syndrome, which particularly accompanies some types of neuroendocrine neoplasia and lung cancers that may produce abnormal ACTH with skin actions [16,17,18,19,20].

#### 2.1.1. Oral Mucosa Pigmentation in AD

**Figure 2:**
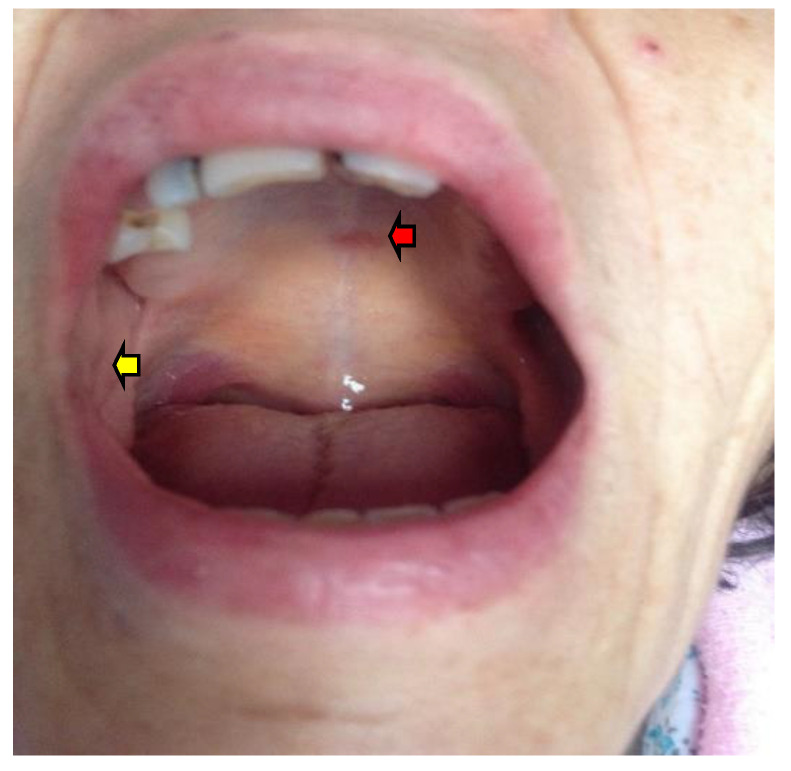
Young adult with Addison disease: clinical aspect of the pigmentary mucosa in the palatine arch in association with dental impressions (red arrow = pigmentary mucosa palatine, yellow arrow = dental impressions). The female patient was already diagnosed with AD with partial compliance with hormonal replacement therapy; oral manifestations were registered while she was under endocrine surveillance.

AD of different etiologies is associated with hyperpigmentation of the skin (mostly at the level of sun-exposed and friction areas, and tissues surrounding scars) as well as mucosa, including oral mucosa [21,22,23] (Figure 2).

Hyperpigmentation is identified in 92% of AD patients; it might be synchronous or precede general clinical manifestations by up to a decade [24]. Biopsy of pigmented lesions in AD shows not only melanocytic infiltration of the basal epidermal layer and melanophages in the superficial dermis, but also acanthosis, perivascular lymphocytic infiltrate, and hyperkeratosis [25]. While the buccal area is the most affected by oral mucosal lesions, the pigmentation may also be located on the palatine arches, lips, gums, and tongue [26,27,28,29,30,31,32,33,34,35].

Pigmented oral lesions vary from a patchy lesion (for instance, macular-like or nodular-like) to a diffuse colored area, underlying various histological types [26,27,28,29,30,31,32,33,34,35]. Different colors are described; the most frequent is dark brown or black [26,27,28,29,30,31,32,33,34,35]. The dark-blue tendency might be misinterpreted as cyanosis [34]. Many cases of oral hyperpigmentation are asymptomatic, but some are accompanied by local pain, itchiness, and burn-like lesions [26,27,28,29,30,31,32,33,34,35]. The combination of color, localization, and surface arrangement represents a useful index of suspicion for AD, especially in previously unrecognized AD cases [26,27,28,29,30,31,32,33,34,35]. It is essential to adequately investigate oral lesions since they are frequently registered before extraoral manifestations of AD, as opposed to skin hyperpigmentation which accompanies general signs and symptoms such as hypotension, nausea, vomiting, anorexia, and weight loss [26,27,28,29,30,31,32,33,34,35].

A tissue biopsy performed by an experienced dermatologist might prove useful in differentiating AD-related lesions from other conditions; yet, biopsy is not routinely indicated [29,30]. Further on, specific endocrine exploration is mandatory for confirmation and adequate therapy of AD in addition to multidisciplinary surveillance. 

Other endocrine conditions displaying mucosal hyperpigmentation that should be differentiated from AD include Cushing’s disease, Graves’ disease, Nelson’s syndrome, and McCune–Albright syndrome, as well as other syndromes with potential endocrine involvement such as Peutz–Jeghers syndrome [33,36,37]. Among nonendocrine conditions, we mention smoker’s melanosis, drug- or tattoo-pigment-associated pigmentation, and Laugier–Hunziker syndrome [33,36,37,38,39,40]. Laugier–Hunziker syndrome represents a rare, noncongenital, benign pigmentation of the oral mucosa, lips, and nails, a condition that is typically a diagnostic of exclusion [38,39,40]. Genetic and inflammatory causes represent endogenous factors of pigmentation, while iatrogenic and environmental exposure is considered an exogenous factor [37]. Some authors reported a parallelism of oral pigmentations with similar lesions on nails [37,39,41].

From a histological perspective, melanocytic lesions, either solitary or multiple, involve a high concentration of reactive melanocytes due to hormonal stimulation, regardless of whether AD is caused by an autoimmune background or not [26,27,28,29,30,31,32,33,34,35]. The oral hyperpigmentation might not regress under glucocorticoid replacements for AD, despite clinical improvement and the control of serum ACTH levels being achieved [26,27,28,29,30,31,32,33,34,35]. Generally, the color of mucosal lesions is also related to nonmelanocyte elements such as vascularization of the adjacent conjunctive tissue and epithelial keratinization [33,42]. Gingival tissues commonly have a pale pink color, although the color may be related to the person’s skin color, tissue thickness, degree of keratinization, and various comorbidities [33,42]. Additionally, the level of circulating hemoglobin may influence the coloration, and it should be noted that AD-related anemia is not uncommon due to a heterogeneous panel of digestive complications; some anemia-related oral anomalies are also detectable before the actual diagnosis of the underlying condition [43,44]. Dantas et al. described the first case of oral multifocal melanoacanthoma (a rare, benign, pigmented lesion, usually involving both melanocytes and keratinocytes) in a 50-year-old patient who was newly diagnosed with synchronous AD and Basedow–Graves disease [31]. The patient was admitted for two pigmented spots at the level of her upper lip mucosa, and further investigations were necessary. Biopsy provided pathological confirmation as well as immunohistochemistry analysis for HMB-45, a promelanosome marker [31]. The lesion was not regressive at the moment the subject was treated for endocrine conditions of both the thyroid and adrenal glands [31]. Melanoacanthoma has a reactive pathogenesis in more than two-thirds of cases underlying different factors [32]. A rapidly growing lesion or the presence of an irregular shape indicates a biopsy [32].

#### 2.1.2. AD-Related Tongue Anomalies of Color

**Figure 3:**
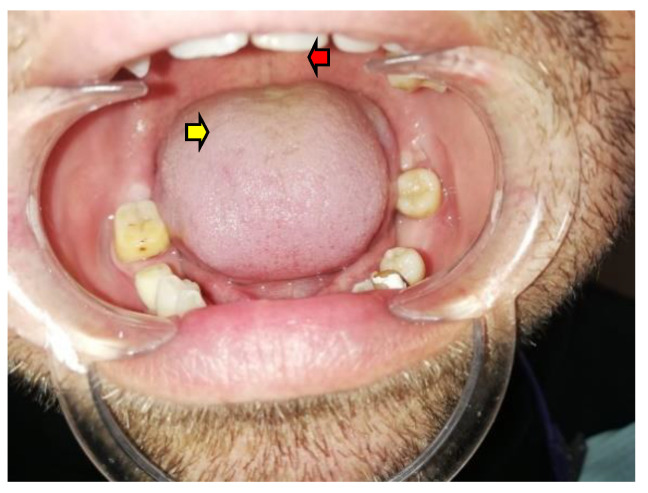
Addison disease with poor control of disease in an adult male: tongue anomalies revealing depapillation with fissured and brown pigmented lingual features (red arrow shows brown pigmented lingual features; yellow arrow shows depapillation of the tongue in addition to fissured lesions). The patient was partially compliant with glucocorticoid substitution and showed poor nutritional status.

Recently, tongue analysis has been the subject of interesting clinical and preclinical studies concerning general autoimmune conditions [45,46]. This muscle represents a frequently neglected subject of examination, yet it has a major role in daily life due to its involvement in eating, speech, and thus in overall QL [45,46]. Tongue evaluation is part of the orofacial assessment in patients with AD on first admission or during follow-up for adrenal disease [45,46,47,48] (Figure 3).

In AD, tongue discoloration is reported sometimes as the first sign of adrenal involvement; thus it is important to adequately investigate a patient who otherwise may be asymptomatic [47]. Seeker and Osswald recently reported a 33-year-old female admitted for tongue hyperpigmentation [47]. After an ACTH stimulation test was performed, AD was confirmed [47]. Even in apparently healthy subjects with an irrelevant general clinical exam and negative medical family history who are admitted for patchy changes in tongue color, the index of suspicion regarding AD should not be dismissed [45,46,47,48]. The pigmented lingual patches may be single or multiple; they are situated at the level of dorsal and lateral areas, either isolated or synchronous with similar lesions at other anatomical parts such as lips and palatine arches [45,46,47,48]. In addition to hormonal imbalance-related pigmentation, fungiform pigmented papillae are reported in AD individuals [49,50,51]. The dermoscopy examination is particularly useful for fungal etiology; yet, it does not represent a popular method of investigation in dental and oral daily practice [50]. In addition, AD represents one of the rarest noncongenital causes of blue tongue, a temporary dyschromia that has been described in relationship with cyanosis or thrombocytosis [52].

### 2.2. Spectrum of AD-Related Comorbidities: Oral Health Perspective

#### 2.2.1. Oral Candidiasis in Patients with APS1

Candidiasis is part of APS1, also called autoimmune polyendocrinopathy–candidiasis–ectodermal dystrophy (APECED), a rare monogenetic condition underlying autoimmune regulator (*AIRE*) gene mutation on chromosome 21q22.3 [53,54,55]. The AIRE protein is responsible for immune self-tolerance which causes immune dysregulation and autoimmune conditions in APS1, typically involving the classical triad: AD, hypoparathyroidism, and chronic cutaneomucosal candidiasis [53,54,55]. Hypoparathyroidism is usually the first endocrine manifestation and the most common condition; moreover, APS1 includes type 1 diabetes mellitus (DM), autoimmune hypothyroidism, premature ovarian failure, and other nonendocrine disorders [56,57].

Chronic mucosal and skin candidiasis represents an early sign of APS1 which is caused by autoantibodies against interleukin (IL)-17A, IL-17F ± IL-22, and probably a high mucosal concentration of IFN-γ; excessive amounts of different autoantibodies represent an effect of defective thymic deletion induced by AIRE-mediated autoreactive T cells [58,59]. The assessment of autoantibodies against IL-17A/F and IL-6 might help in early clinical diagnosis [60].

Oral candidiasis must be recognized in relationship with APS-related adrenal insufficiency, especially in the pediatric population, since the syndrome onset typically occurs during childhood with a progressive risk of multiple organ involvement [54,61]. One particular clue before the actual endocrine diagnosis might be recurrent oral thrush [62]. Atypical presentations with adult onset of APS-related mucocutaneous candidiasis are reported; this rare syndrome represents a challenge in the adult population as well [63,64,65]. Tenório et al. recently reported a 42-year-old female diagnosed with chronic hyperplastic candidiasis, including angular cheilitis, in association with AD and hypoparathyroidism as part of APS1 [53]. She also presented microstomia, xerostomia, and dental anomalies such as hypoplastic teeth [53]. Local and/or systemic therapy with antifungal medication is useful in addition to careful hormonal substitution for AD [53].

A study published in 2021 on 938 cases of APECED showed the following: 57% of cases had classical triad; candidiasis was the earliest sign in 82% of subjects; hypoparathyroidism was the most frequent disorder (84%) while AD was the latest manifestation (72%); and 58% of patients had different infections, with 5% of them being adults at first APS1 diagnosis [66]. Another survey published in 2021 on 158 patients with APS1 who were followed for 23.7 ± 15.1 years showed that APS1 prevalence was 2.6 cases per 1 million, and the prevalence of AD, hypoparathyroidism, and chronic candidiasis was 77%, 86%, and 74%, respectively [67]. We also mention a prevalence of 29% for autoimmune intestinal disorders, 25% for pernicious anemia with atrophic gastritis, 17% for vitiligo, and 2.5% for celiac disease, all of these comorbidities with other potential oral manifestations [67].

The long-term evolution of untreated or suboptimally treated mucocutaneous candidiasis can lead to systemic candidiasis or even oral squamous cell carcinoma, chronic candidiasis being considered a procarcinogenic status in APS1 as suggested by some authors, based on Th17 cell anomalies [68,69].

Overall mortality is increased in APS1, and prompt treatment of each endocrine component, especially AD, and infections is mandatory [70,71]. A Finnish study on 91 patients with APS1 (between 1971 and 2018) confirmed the overall increased mortality, as expressed by a standardized mortality ratio of 11 (95% confidence interval between 7.2 and 16, *p* < 0.001) [71]. A cohort study on deceased patients with APS1 (between 1970 and 2019) showed that the most common cause of death was infections in 24% of the cases, followed by oral and esophageal cancers in 15% of them, followed by disturbances of the circulatory system with potential contribution of an adrenal crisis [70].

We also mention that diabetic patients (including APS-related DM) are prone to opportunistic infections such as oral candidiasis; the production of different exoenzymes such as phospholipases, hemolysinases, and esterase explains various virulence profiles among Candida species [72,73]. A study from 2021 on diabetic subjects with oral candidiasis identified 108 Candida species (75 of Candida albicans with the most powerful exoenzyme activity and 33 not of Candida albicans type) [72]. Generally, oral candidiasis may remain a superficial condition, or it might become invasive with multiorgan dissemination, especially in immunocompromised patients as seen in AD, APS1, and uncontrolled DM ± AD/APS [72,73]. The spectrum of effective antimycotic drugs includes nystatin, amphotericin, and miconazole; some of them might impair steroidogenesis through cytochrome 450 activity and precipitate an acute adrenal crisis in a previously unrecognized AD patient [72,73].

#### 2.2.2. Early-Onset APS-Related Hypoparathyroidism and Dental Anomalies

As mentioned, autoimmune hypoparathyroidism is the earliest endocrine dysfunction in APS1, and thus children might experience the dental complications of persistent low parathormone (PTH) and low calcium levels [74,75,76]. Short dental roots, a challenge for orthodontists, with or without enamel hypoplasia/dysplasia are mostly reported [74,75,77]. These may be identified before the actual diagnosis of autoimmune AD [74,75]. While oral candidiasis is reported before the age of 5 in APS1 cases, enamel hypoplasia is typically registered before the age of 15, and thus the presence of chronic candidiasis in a child with dental enamel hypoplasia represents a warning sign for autoimmune hypoparathyroidism [74]. A systematic review on nonsurgical hypoparathyroidism and pseudohypoparathyroidism included 88 studies (9 transversal studies, 1 prospective study, 26 case series, and 55 case reports); the research revealed the most frequent dental anomalies in patients with low PTH are enamel hypoplasia, enamel opacities, hypodontia, and eruption anomalies [78]. Other dental anomalies that have been reported in hypoparathyroidism include delayed tooth eruption, poorly calcified dentin, and anomalies of dental development [79,80,81]. All mentioned dental manifestations are part of the more complex picture of oral manifestations in APS1 patients which includes not only chronic oral candidiasis and ACTH-related mucosal pigmentations, but also general symptoms/signs of chronic hypocalcemia such as paresthesia of the tongue and lips and facial muscle cramps (as seen in other body parts) [79,80,81].

#### 2.2.3. From AD-Related type 1 DM to Periodontal Disease

AD is associated with type 1 DM in APS2 and rarely in APS1; the most common autoimmune associations of type 1 DM are AD and autoimmune diseases of the thyroid, and it has been noted that 8% of the general population suffers from an autoimmune disease [82,83,84,85]. A registry-based study on type 1 DM showed that patients with associated AD and/or antibody-induced thyroid diseases require higher insulin doses than patients with isolated type 1 DM [85]. Concerning DM and AD, both conditions involve abnormal T-cell behavior [82,83,84]. While no particular studies address the issue of periodontal disease (PD) in AD subjects, PD is essentially important in diabetic patients, and thus PD should be particularly taken into consideration in the diabetic subgroup of individuals with AD [86,87]. Currently, there is a very large number of studies targeting the topic of PD in the diabetic population as it represents a major health concern; for instance, a meta-analysis from 2022 identified 2151 scientific papers regarding DM–PD [88]. DM and PD are linked to chronic inflammation; adequate control of glycemia improves PD [89,90].

Generally, severe periodontal inflammation or bleeding requires prompt investigation of conditions such as DM, human immunodeficiency virus infection, thrombocytopenia (which also has been reported in autoimmune AD individuals due to similar antibodies), and leukemia [91,92]. As mentioned, DM causes anomalies of the oral microbiome at subgingival and salivary levels [93,94,95]. However, PD represents a multifactorial entity that is typically triggered by an infection with Gram-negative bacteria (for instance, Bacteroides forsythus, Porphyromonas gingivalis, and Prevotella intermedia), thus contributing to the onset of a local, progressive immune response based on the main features of bacteria: invasiveness and toxigenicity [96,97,98,99,100]. The bacterial cells interfere with periodontal cells through exotoxins, enzymes, and metabolites; then, exotoxins and leukotoxins interfere with polymorphonuclear leukocytes (PMNs), destroying the leukocytes in the gingival ditch and causing the colonization and invasion of the periodontal tissue [96,97,98,99,100]. Inflammatory mediators act on gingival conjunctive tissue, the periodontal ligament, and the alveolar bone [96,97,98,99,100]. Marginal periodontitis is triggered and supported by the activity of microorganisms within the bacterial plaque; still, the single presence of microorganisms is not the exclusive factor responsible for PD [97,101]. Porphyromonas gingivalis, for instance, was frequently associated with various endocrine conditions such as DM, obesity, and acromegaly [97,98,99,100]. This bacteria is able to disseminate within the peripheral blood flow, causing general inflammation [96,97,98,99,100,101,102]. Chronic periodontal inflammation induces the destruction of periodontal ligaments and alveolar bone, with consecutive tooth loss [103,104,105]. The covering periodontium (gingival fibromucosa) is affected, as is the support or apical periodontium, which is made of cementum, alveolar bone, and periodontal ligament [106,107,108]. An important role in the onset of endocrine periodontopathy is played by not only local factors within the oral cavity, but also an abnormal immune response at the level of periodontium [109,110]. Bacteria in the subgingival biofilm cause the migration of PMNs from the gingival ditch into the tissues, with the consecutive release of chemotactic factors for the circulating neutrophils [111,112,113,114,115,116,117]. Macrophages play an important role due to their main function, namely phagocytosis of pathogenic germs and cellular debris, and subsequently present the antigen information to B and T lymphocytes, triggering the adaptive specific immune response; macrophages also release cytokines such as IL-1, IL-17, tumor necrosis factor α (TNF), leukotrienes, and prostaglandins with a role in tissue destruction, finally causing alveolar bone resorption [111,112,113,114,115,116,117]. In the presence of a bacterial infection, PMNs release a high amount of metalloproteinase (MMP) in an attempt to neutralize the pathogens invading the periodontium [111,112,113,114,115,116,117]. Overproduction of MMPs leads to collagen (the main component of the periodontium) being damaged; thus, PMNs, which normally play a part in the organism’s defense, are the factor deteriorating the periodontium [111,112,113,114,115,116,117]. MMPs are endopeptidase enzymes, proteases with a role in the extracellular matrix, and are released by the epithelial keratinocytes, fibroblasts, macrophages, and leukocytes [111,112,113,114,115,116,117]. In chronic marginal periodontitis, levels of MMPs (for example, MMP-2, MMP-8, and MMP-9) are higher than those in the healthy periodontium, and these high levels are associated with irreversible tissue damage and periodontitis progression; MMP-2 and MMP-9 may damage collagen type IV of the basal lamina and other matrix proteins, such as collagen types V and VII, fibronectin, laminin, and elastin, while MMP-8 destroys collagen types I, II, III, and VII [111,112,113,114,115,116,117]. Alterations of immune status are found in AD and DM patients, while generally both pathologies are associated with a higher risk of infections, especially in patients with suboptimal glucocorticoid replacement and patients with high levels of advanced glycation end-products (AGEs) [118]. Half of the acute presentations concerning AD are related to an infection that requires increasing glucocorticoid doses or switching to intravenous regimes, indicating that these individuals are more exposed to chronic complications as well, including infections and/or oral manifestations [119].

#### 2.2.4. Anemia in AD Individuals

Anemia in AD has multifactorial mechanisms, clinically being manifested with oral mucosal pallor, among other manifestations; the panel of AD comorbidity-related anemia varies from endocrine disorders (autoimmune hypogonadism, DM, autoimmune hypothyroidism/myxoedema) to a heterogeneous group of nonendocrine autoimmune conditions [120,121]. Rheumatologic diseases such as lupus erythematosus and systemic sclerosis involve particular oral features; for instance, lupus induces ulcerations, honeycomb plaques, keratotic plaques, angular cheilitis, buccal petechial lesions, and a higher risk of periodontitis [120,121,122,123,124,125,126]. Gastroenterological conditions such as Crohn’s disease, ulcerative colitis, and gastritis have various oral anomalies; Crohn’s disease presents oral ulcerations, mucogingivitis, and a 2–3 times higher risk of PD than the general population [127,128,129,130]. One systematic review of six studies showed that patients with inflammatory bowel disease, either Crohn’s disease or ulcerative colitis, had a decayed, missing, and filled teeth (DMFT) index) 3 times higher than that of the healthy population, which is suggestive of a more frequent past and present diagnosis of dental caries [129]. Autoimmune atrophic gastritis, more frequent in adults than in the pediatric population, requires a complex gastroenterological evaluation, even a gastric biopsy, as well as adequate replacements with vitamin B12; it is caused by antibodies against parietal cells and intrinsic factor [131,132]. The underlying vitamin B12 deficiency of Biermer’s disease might cause hyperpigmentation by itself, mimicking AD-associated hyperpigmentation [133]. Regardless of the presence of clear criteria for APS, individuals with gastric antibodies may present similar antibody-related conditions such as Hashimoto thyroiditis and myasthenia gravis and dermatologic disorders such as chronic urticaria, vitiligo, and lichen planus, all of these comorbidities with a heterogeneous potential for oral mucosa anomalies [132].

#### 2.2.5. Oral Features of Other Non-DM, Autoimmune Comorbidities in AD Patients

Hashimoto thyroiditis accompanies autoimmune AD in APS2 (Schmidt syndrome), and rarely in APS1; generally, it is considered that autoimmune thyroid disease is the most frequent autoimmune condition associated with AD [134,135,136]. APS2 is a polygenic condition traditionally related to the human leukocyte antigen (HLA) complex on chromosome 6; APS2 includes AD and autoimmune hypothyroidism, as well as other endocrine disorders such as DM and premature ovarian failure [134,135,136]. A nationwide registry-based study published in 2022 including 912 subjects with autoimmune AD confirmed that 48% of them had an autoimmune thyroid disease (20% with hypothyroidism, 73% with subclinical hypothyroidism) as the most frequent endocrine comorbidity [137]. Congenital hypothyroidism and severe forms of hypothyroidism with early onset during childhood are associated with oral manifestations such as macroglossia, micrognathia, thick lips, enamel hypoplasia, and delayed tooth eruption, a picture which is less likely to be manifested in AD-associated autoimmune thyroid dysfunction [138]. A study from 2021 on 635 patients with hypothyroidism, mostly of autoimmune cause, who received 1480 implants between 2000 and 2017 showed that peri-implant bone loss was statistically significantly lower than in patients with normal thyroid function, suggesting that the condition is not associated with a higher risk of dental implant failure, probably via lower metabolic rate [139].

A high rate of vitamin D deficiency (VDD) is reported among AD individuals; for instance, a study from 2022 on five European cohorts, a total of 1028 patients with autoimmune AD, found that 34–57% of them had VDV, defined as a level of 25-hydroxyvitamin D (25OHD) between 10 and 20 ng/mL, while 5–22% had severe VDV (a value of 25OHD below 10 ng/mL) [140]. VDV impacts general oral health, especially in children, pregnant women, and seniors (where reports showed correlations with bone density); it is suggested to be an additional contributor to PD, and probably to dental implant failure [141,142,143]. VD replacement might help the process of osseous integration after dental implants, as its role in bone healing has been noted [144,145].

Malabsorption in addition to celiac disease (CD) has been reported in AD patients; CD is an immune-mediated small intestinal disease affecting both children and adults, with a general prevalence in the general population of 0.5–1%; the diagnosis is based on clinical, serological assessments and small intestinal biopsies [146,147,148]. CD is associated with other autoimmune diseases: most often type 1 DM in children and teenagers and autoimmune thyroid disorders (mostly hypothyroidism) in adults, but also autoimmune types of AD, hepatitis, hypoparathyroidism, hypopituitarism, premature ovarian failure, etc. [147,149]. The enteropathy triggered by the ingestion of gluten is correlated with various oral manifestations such as xerostomia, dry lips, mucosal ulcerations, and sialadenitis [150]. A recent pediatric study confirmed that children with CD have a higher rate of recurrent aphthous stomatitis and dental enamel defects than the healthy population [151]. Moreover, CD is significantly associated with a higher prevalence of caries and dentin sensitivity, while an adequate gluten-free diet controls gingival bleeding [152].

Vitiligo has been reported in association with autoimmune AD, as well as other endocrine conditions, especially conditions of the thyroid, type 1 DM, etc. [153,154,155]. Vitiligo-associated depigmentation affects both teguments and mucosa, including the oral cavity [155,156]. One study on 100 patients diagnosed with vitiligo showed that 5% of them had buccal mucosa involvement, 8% had palatal lesions, 2% had gingival depigmentation, and 1% had alveolar mucosa anomalies, while lips (one or both) were affected in 42% of cases, with most of these subjects having associated facial extension [156]. Overall, more than half of the patients diagnosed with vitiligo had oral involvement of a different form [156].

#### 2.2.6. Exceptional Entities of AD with Potential Oral Manifestations

Triple-A syndrome (also named Allgrove syndrome), another autosomal recessive disorder involving primary adrenal insufficiency (due to an ACTH resistant status), is a very rare condition with underlying mutations of the AAA (or AAAS) gene on chromosome 12q13 in 90% of cases; it also includes alacrimia (defects of tear formation) and (esophageal) achalasia, which induces difficulties of swelling, dry mouth, and vomiting due to esophagitis with potential dental damage [157,158,159,160]. Tadini et al. reported a case of triple-A syndrome associated with peculiar dental anomalies [161].

Histoplasmosis, a common fungal infection caused by the fungus Histoplasma capsulatum, may cause both oral lesions and, rarely, dissemination to adrenals inducing AD [162,163,164]. The oral mucosa may be affected at any level, mostly being affected by tongue, buccal, and palatal ulcers with local pain, lesions that frequently cannot be distinguished from a malignant lesion unless a biopsy is performed [162]. A particular presentation is acquired perforating dermatosis with underlying damage to dermal proteins with pruritic nodules and hyperpigmentation [163]. Antifungal therapy is mandatory and cortisol replacement is lifesaving in adrenal histoplasmosis [163].

### 2.3. Oral Surgery Procedures in Patients with AD

Oral surgery procedures such as surgical third-molar removal, implant surgery, maxillary bone augmentation, and mucogingival surgery require higher doses of hydrocortisone replacement or intravenous supplementation with close control of blood pressure in AD subjects [165,166,167]. On the other hand, there is the general issue of dental implants in patients with different autoimmune disorders. While no particular guidelines address this issue, most studies agree that the implant survival rates seem similar to those of the healthy population; however, the management of implant prosthetic treatment and follow-up needs to be adjusted depending on the disease [168,169,170].

Although limited data are specifically provided for AD individuals, the new implant techniques seem safe for a patient under adequate glucocorticoid replacement in addition to strict oral hygiene and serial check-up [171]. Another concern is related to a potential adrenal crisis in a dental patient. One review of the literature identified 148 articles concerning this particular matter and selected 34 papers for final analysis [172]. The results showed that an acute adrenal insufficiency under these circumstances is mostly triggered by pain, local infection, insufficient hormonal replacement, poor general health status, an invasive dental procedure, and the use of a barbiturate as a general anesthetic, and the risk of crisis was estimated by authors to be 1 in 650,000 AD patients [172]. Dental health practitioners must take into consideration the special needs of an AD patient, and they should be aware of particular orodental aspects in these subjects [173,174].

Generally, a patient with AD is more prone to develop any type of infection, including in the oral area, which requires prompt recognition and specific anti-infectious therapy and adjustment of hydrocortisone substitution [175,176]. Patient education is essential for good long-term management, including dose adjustment to daily stress; the indication for switching the oral regime; and the recognition of infections and other general health issues, including dental health, that might interfere with glucocorticoid replacement [175,177].

## 3. Conclusions

It is essential to adequately investigate oral pigmented lesions since they are frequently registered before extraoral manifestations of AD, as opposed to skin hyperpigmentation which accompanies general signs and symptoms. An oral lesion might provide a useful index of suspicion in apparently healthy individuals. The spectrum of autoimmune/non-autoimmune comorbidities massively complicates the overall picture of oral manifestations in AD, and thus awareness is important. The key operative strategy under these circumstances is a multidisciplinary team, from the first diagnosis of an AD-associated oral lesion to the entire period of endocrine surveillance.

## Data Availability

Not applicable.

## Abbreviations

ACTH	adrenocorticotropic hormone
AGEs	advanced glycation end-products
AD	Addison disease
APS	autoimmune polyglandular syndrome
APECED	autoimmune polyendocrinopathy–candidiasis–ectodermal dystrophy
CD	celiac disease
DM	diabetes mellitus
DMFT index	decayed, missing, and filled teeth index
HLA	human leukocyte antigen
25OHD	25-hydroxyvitamin D
IL	interleukin
MMP	metalloproteinase
MSH	melanocyte stimulating hormone
MCR	melanocortin receptor
PD	periodontal disease
POMC	proopiomelanocortin
PMN	polymorphonuclear leukocyte
PTH	parathormone
TNF	tumor necrosis factor
QL	quality of life
VDD	vitamin D deficiency
